# Errata

**Published:** 1887-01

**Authors:** 


### ERRATA.

Pape 410, line 16: for *properly* read *profusely*; line 17: for P. M. read A. M.

